# Criticality Assessment Method for Automated Driving Systems by Introducing Fictive Vehicles and Variable Criticality Thresholds

**DOI:** 10.3390/s22228780

**Published:** 2022-11-14

**Authors:** Demin Nalic, Tomislav Mihalj, Faris Orucevic, Martin Schabauer, Cornelia Lex, Wolfgang Sinz, Arno Eichberger

**Affiliations:** 1 ADAS Department, MQS Automotive AT OG, 8074 Raaba, Austria; 2 Institute of Automotive Engineering, Graz University of Technology, 8010 Graz, Austria; 3 ADAS Simulation and Data Analysis Department, MAGNA Steyr Fahrzeugtechnik AG Co. & KG, 8041 Graz, Austria

**Keywords:** fictive vehicles, safety assessment, scenario criticality, automated driving

## Abstract

The safety approval and assessment of automated driving systems (ADS) are becoming sophisticated and challenging tasks. Because the number of traffic scenarios is vast, it is essential to assess their criticality and extract the ones that present a safety risk. In this paper, we are proposing a novel method based on the time-to-react (TTR) measurement, which has advantages in considering avoidance possibilities. The method incorporates the concept of fictive vehicles and variable criticality thresholds (VCTs) to assess the overall scenario’s criticality. By introducing variable thresholds, a criticality scale is defined and used for criticality calculation. Based on this scale, the presented method determines the criticality of the lanes adjacent to the ego vehicle. This is performed by placing fictive vehicles in the adjacent lanes, which represent copies of the ego. The effectiveness of the method is demonstrated in two highway scenarios, with and without trailing vehicles. Results show different criticality for the two scenarios. The overall criticality of the scenario with trailing vehicles is higher due to the decrease in avoidance possibilities for the ego vehicle.

## 1. Introduction

The assessment of ADS is essential for the approval and testing procedures. Due to a high scenario space and the complexity of ADS that needs to be tested for safety approval, scenario-based methods are promising and widely used approaches in the field of research. Recent studies, for example, [1,2], are focused on simulation-based testing and the generation of scenarios to reduce the testing effort for ADS. The reduction is achieved by identifying relevant safety-critical scenarios (SCS) using a variety of safety metrics and scenario-generation methods. A comprehensive review of current studies related to scenario-based approaches is presented in [3]. A scenario in the context of this work represents a series of actions occurring over a period of time, as defined in [4]. Other similar definitions are introduced and can be found, for example, in [5,6]. After extracting scenarios from a synthesized or real dataset, the scenarios are analyzed and assessed on their criticality. There are different definitions of what the criticality of a certain situation means. According to [7], accident probability combined with severity defines the criticality of a certain situation. The probability which is used here takes into account the avoidance of a particular situation. This approach enables a better interpretation of a specific situation. To calculate accurate avoidance possibilities, environment parameters, which are composed of static and dynamic objects, should be considered for evaluating the criticality of the situation. The available probabilistic methods, such as those in [8,9,10], calculate possible evasive maneuvers or accident probabilities; based on these, they estimate whether a situation is evasive, critical, or fulfills safety criteria. This consideration is advantageous on the one hand because it takes into account a large number of possibilities and the probability or feasibility of these possibilities in the calculation. On the other hand, due to complex solutions to mathematical problems, real-time capabilities and unique solutions are only sometimes given. To overcome such issues, we introduce a novel assessment method that introduces fictive ego vehicles on the left and right lanes adjacent to the tested ego vehicle. These fictive ego vehicles represent the exact copy of the ego vehicle, i.e., the vehicle configuration and the automated driving functions are the same. In this constellation, the ego vehicle and the neighboring fictive ego vehicles are assessed individually in the respective lane according to the introduced criticality scale. Combining the criticalities of the ego and fictive vehicle, an overall criticality of the situation can be calculated. To calculate the criticality, we take the avoidability of a collision into account using the TTR metric. The TTR metric consists of the time-to-brake (TTB) and time-to-steer (TTS) measurements, which represent the times left to avoid a critical situation by braking or steering, respectively. For SCS assessment, usually, the fixed metric thresholds are defined. However, metrics often depend on the kinematic relations between the ego and surrounding objects, such as distances and velocities. Although time-based metrics capture the influence of both distance and velocities, thresholds remain changeable depending on the object states. To overcome this issue with TTR, we are introducing variable criticality thresholds (VCTs) that determine a criticality scale. Finally, combining the concept of fictive ego vehicles, the TTR, and the VCTs, we derived the novel assessment method presented in this work. In the first Section 2, we present a detailed analysis of safety metrics used in scenario-based approaches for the assessment of SCS and a detailed description of the usage of the TTR. Using the TTR the criticality definition and the assessment method are presented in Section 3. As the last step of the work, we demonstrate the effectiveness of the presented method based on a safety-relevant test example.

## 2. Safety Metrics

According to [11], safety metrics are classified into temporal-, distance- and deceleration-based safety metrics. In [12], additional categorizations are introduced: the statistics-based, potential-field-based, and unexpected-driving-behavior-based metrics. These reviews show that temporal safety metrics are often and widely used to assess scenarios. The leading representative that is often mentioned in the context of the criticality of scenarios is the TTC. The main advantage of the TTC lies in its simplicity and efficiency for rear-end scenarios. The main disadvantages of the TTC criteria are the insensitivity to lateral collision risks and the fact that it cannot reflect the potential risk when the relative speed between two vehicles is zero. This implies that a possible intervention of a driver in safety-critical situations is not taken into account by the TTC. Therefore, various adjustments and adaptations of the classic TTC time were made; for example, a modified time-to-collision (MTTC) measure was introduced in [13]. Compared with the conventional TTC, the MTTC considers relative acceleration and relative velocity to improve collision prediction. In [14], the worst-time-to-collision (WTTC) metric was introduced. The WTTC considers the influence of vehicle dynamics and avoidance possibilities of the ego vehicle with the worst-case assumption regarding vehicle behavior. Another approach based on the TTC is presented in [15], where a time-exposed TTC (TET) and the time-integrated TTC (TIT) are introduced. These two metrics are extensions of the TTC and take the whole trajectory of vehicles over space and time into account. In [16], the TTR was introduced for the development and evaluation of forward-collision-avoidance systems, which consider a TTS, a TTS, and a TTK. In general, these reaction times determine the remaining time for successful collision avoidance by braking, steering, or acceleration. A similar approach is described in [17]; here, in addition to the TTS, a requested acceleration is introduced and used for the assessment of certain scenarios for ADS. The main advantages of these approaches compared with conventional temporal metrics such as the TTC and extensions of the TTC [18,19] are in consideration of avoidance possibilities and kinematic behavior of vehicles. The later the driver reacts successfully to an impending collision, the more the system is forced to exploit the vehicle’s physical limits. Using the TTR as a safety metric offers the possibility to interpret and define the criticality in a wide range of safety-relevant scenarios. The present work represents a continuation of previous studies of [16,17,20], where longitudinal and lateral kinematics are taken into account for a criticality assessment of ADS.

**Figure 1:**
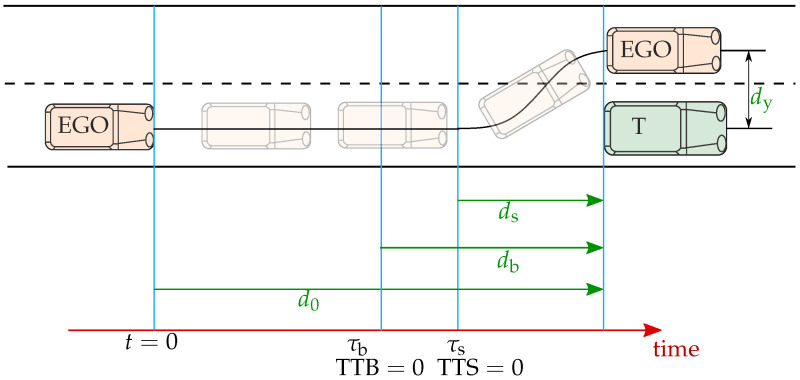
Visualization of steering and braking possibilities.

**Figure 2:**
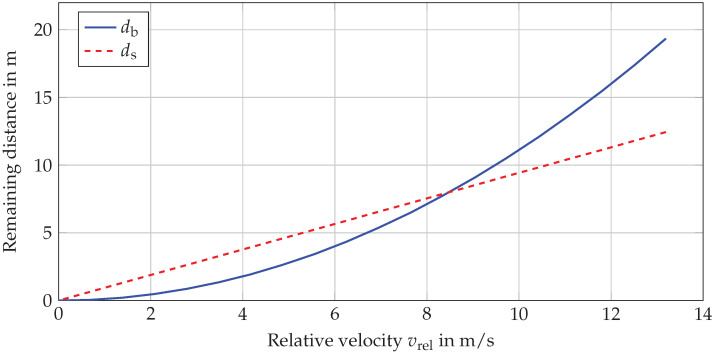
Remaining distance of an ego vehicle to a target vehicle for avoiding a rear-end collision in longitudinal traffic for a pure braking maneuver and a pure steering maneuver.

Time-to-react is defined as TTR = max{TTB, TTS}, where TTB and TTS are the time-to-brake and time-to-steer metrics, respectively. They represent time reserves to the last point to brake (LPTB) and last point to steer (LPTS) [21]. The points at which the ego has to perform braking or steering to avoid a collision with a vehicle in front, also called a target vehicle. For practical reasons, the times at which TTB and TTS are equal to 0 will be denoted as $\tau_{b}$ and $\tau_{s}$, respectively. In their general and simplest forms, they are defined by (1) and (2). The remaining distances between the ego and the target vehicle at $\tau_{b}$ and $\tau_{s}$ are defined as $d_{b}$ and $d_{s}$ by (3) and (4), respectively. The $d_{b}$ is the distance that ego travels while reducing its velocity ($v_{\mathrm{ego}}$) to the target’s velocity $v_{t}$ by applying the maximum braking $a_{\min,x}$ [21]. It is worth mentioning that $a_{\min,x}$ stands for the maximum longitudinal deceleration, wherein we respect the positive and negative signs of acceleration in the longitudinal direction. Here, $d_{s}$ is calculated as equivalent to the evasion time $t_{\mathrm{ev}}$ (5) needed to change a lane with maximum lateral acceleration, $a_{\max,y}$ [17,21]. Unlike the longitudinal direction, in the lateral direction, we do not consider negative signs; therefore, $a_{\max,y}$ denotes the maximum acceleration, regardless of direction. These temporal and spatial relations are depicted in Figure 1, while all the parameters used to calculate the metrics and their thresholds are summarized in Table 1. Figure 1 illustrates that $\tau_{s}$ occurs at a later point in time than $\tau_{b}$, which indicates the steering as the last emergency intervention to avoid a collision; however, this is not always the case. Figure 2 shows the dependency of the remaining distances $d_{b}$ and $d_{s}$ on the relative velocity between the ego vehicle and target vehicle. Here, braking leaves more time to avoid a collision at low velocities, but at higher relative velocities, steering would be more appropriate.

(1) $$\begin{array}{c} \tau_{b}=\frac{d_{0}-d_{b}}{v_{\mathrm{ego}}-v_{t}} \end{array}$$

(2) $$\begin{array}{c} \tau_{s}=\frac{d_{0}-d_{s}}{v_{\mathrm{ego}}-v_{t}} \end{array}$$

(3) $$\begin{array}{c} d_{b}=-\frac{v_{\mathrm{rel}}^{2}}{2\;a_{\min,x}} \end{array}$$

(4) $$\begin{array}{c} d_{s}=\sqrt{\frac{2\;d_{y}}{a_{\max,y}}}\;v_{\mathrm{rel}} \end{array}$$

(5) $$\begin{array}{c} t_{\mathrm{ev}}=\sqrt{\frac{2\;d_{y}}{a_{\max,y}}} \end{array}$$

**Table 1:** Parameters used for safety metrics and thresholds.

State parameters	Description
$v_{\mathrm{ego}}$	Velocity of the ego vehicle
$v_{t}$	Velocity of the target vehicle
$v_{\mathrm{rel}}$	Relative velocity between the ego vehicle and the target vehicle in front
$a_{\mathrm{ego},x}$	Current longitudinal acceleration of the ego vehicle
$a_{\min,x}$	Maximum longitudinal deceleration
$a_{\max,y}$	Maximum lateral acceleration
$a_{\mathrm{req},x}$	Required longitudinal deceleration of the ego vehicle
$a_{\mathrm{req},y}$	Required lateral deceleration of the ego vehicle
**Time parameters**	
$\tau_{b}$	Time to reach LPTB (TTB =0)
$\tau_{s}$	Time to reach LPTS (TTS =0)
$\tau_{b,\mathrm{th}}$	Threshold for the $\tau_{b}$
$\tau_{s,\mathrm{th}}$	Threshold for the $\tau_{s}$
$t_{\rho }$	Response time of the ego vehicle
$t_{\mathrm{ev}}$	Evasion time needed to change a lane
**Distance parameters**	
$d_{0}$	Initial distance between the ego and target vehicle at the point t=0
$d_{b}$	Distance to target vehicle at $\tau_{b}$
$d_{s}$	Distance to target vehicle at $\tau_{s}$
$d_{y}$	Lateral evasion distance
$d_{b,\min}$	Minimum safety distance for braking maneuver
$d_{s,\min}$	Minimum safety distance for steering maneuver
**Remaining parameters**	
*g*	Gravitational constant
μ	Road friction coefficient

The previously defined relations in (1)–(4) consider only the constant velocities of the ego and target vehicles representing only a special case and resulting in a reduced precision in the presence of variable velocities. To increase the prediction precision while keeping it feasible regarding its complexity, a straight road and constant velocity $v_{t}$ is assumed, while the ego vehicle keeps constant longitudinal acceleration $a_{\mathrm{ego},x}$ up to the point where evasive braking or evasive steering actions must be executed. Furthermore, considering ideal conditions, the possible maximum braking and lateral acceleration are $a_{\min,x}=-\mu \;g$ and $a_{\max,y}=\mu \;g$, respectively, where μ represents the maximum available tire road friction. Considering such steady states, the remaining distances $d_{b}$ and $d_{s}$ are given by (6) and (7):

(6) $$\begin{array}{c} d_{b}=-\frac{{\left(v_{\mathrm{rel}}+a_{\mathrm{ego},x}\;\tau_{b}\right)}^{2}}{2\;a_{\min,x}} \end{array}$$

(7) $$\begin{array}{c} d_{s}=\sqrt{\frac{2\;d_{y}}{a_{\max,y}}}\left(v_{\mathrm{rel}}+a_{\mathrm{ego},x}\;\tau_{s}\right), \end{array}$$

while $\tau_{b}$ and $\tau_{s}$ are determined by solving the following two quadratic equations.

(8) $$\begin{array}{c} \frac{1}{2}\left(a_{\mathrm{ego},x}-\frac{a_{\mathrm{ego},x}^{2}}{a_{\min,x}}\right)\tau_{b}^{2}+\left(v_{\mathrm{rel}}-\frac{v_{\mathrm{rel}}\;a_{\mathrm{ego},x}}{a_{\min,x}}\right)\tau_{b}-d_{0}-\frac{v_{\mathrm{rel}}^{2}}{2\;a_{\min,x}}=0 \end{array}$$

(9) $$\begin{array}{c} \frac{1}{2}\;a_{\mathrm{ego},x}\;\tau_{s}^{2}+\left(v_{\mathrm{rel}}+a_{\mathrm{ego},x}\;\sqrt{2\;\frac{d_{y}}{a_{\max,y}}}\right)\tau_{s}-d_{0}+v_{\mathrm{rel}}\;\sqrt{2\;\frac{d_{y}}{a_{\max,y}}}=0 \end{array}$$

The metrics described by (6)–(9) are used below to establish the thresholds and assess criticality.

## 3. Assessment Method

### 3.1. Concept of Fictive Vehicles

**Figure 3:**
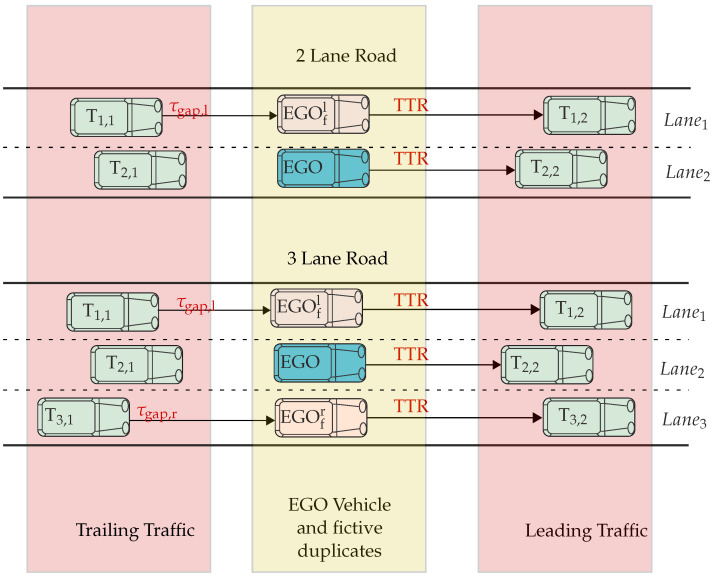
Graphical representation of the two-lane and three-lane scenarios.

Figure 3 shows the novel concept of fictive vehicles on vehicle configurations taken from PEGASUS [22,23], where nine vehicles from the immediate surrounding of the ego vehicle are considered as safety relevant for scenario development or analysis. Furthermore, the vehicles ${\mathrm{EGO}}_{f}^{r}$ and ${\mathrm{EGO}}_{f}^{l}$ represent fictive duplicates of the ego vehicle and are needed for the assessment method described below. However, there is one condition under which a fictive vehicle can be placed in adjacent lanes—the spaces at the neighboring lanes next to the ego must be unobstructed. For better interpretability, the environment around the ego vehicle is divided into trailing traffic, leading traffic, and the part adjacent to the ego vehicle, which consists of fictive vehicles.

### 3.2. Definition of the Criticality

The criticality definition consists of two parts. The first part deals with the definition of variable criticality threshold (VCT) based on the metrics introduced in Section 2. The second part deals with the quantification of the criticality based on the VCT.

#### 3.2.1. Definition of Variable Criticality Thresholds

To determine the VCT, we build upon the required accelerations $a_{\mathrm{req},x}$ and $a_{\mathrm{req},y}$ for braking and steering, respectively, as well as on minimum safety distances for braking ($d_{b,\min}$) and steering ($d_{s,\min}$). Distances are calculated based on the mathematical model of responsibility-sensitive safety (RSS) [24], that accounts for the worst-case scenario, including the response time of the ego and the maximum theoretical braking to the standstill of a target vehicle. However, we introduced slight modifications for calculating $d_{b,\min}$, which are shown in (10). Unlike the RSS, we do not consider the maximum longitudinal acceleration of the ego during the response time; therefore, we use the current ego’s acceleration instead. Another adjustment is regarding the ego’s deceleration after the response time, where we use $a_{\mathrm{req},x}$, which denotes braking but is not limited only to small values.

(10) $$\begin{array}{c} d_{b,\min}=v_{\mathrm{ego}}\;t_{\rho }+\frac{1}{2}\;a_{\mathrm{ego},x}\;t_{\rho }^{2}-\frac{{\left(v_{\mathrm{ego}}+t_{\rho }\;a_{\max,\mathrm{acc}}\right)}^{2}}{2\;a_{\mathrm{req},x}}+\frac{v_{t}^{2}}{2\;a_{\min,x}} \end{array}$$

The safety distance for steering, $d_{s,\min}$, is determined in a similar manner (11). The only difference is that the braking distance of the ego is substituted with the required distance to perform a lane change.

(11) $$\begin{array}{c} d_{s,\min}=v_{\mathrm{ego}}\;t_{\rho }+\frac{1}{2}\;a_{\mathrm{ego},x}\;t_{\rho }^{2}+\sqrt{2\;\frac{d_{y}}{a_{\mathrm{req},y}}}\;\left(v_{\mathrm{ego}}+a_{\mathrm{ego},x}\;t_{\rho }\right)+\frac{v_{t}^{2}}{2\;a_{\min,x}} \end{array}$$

Substituting the initial distance $d_{0}$ in (8) and (9) with the minimum distances $d_{b,\min}$ and $d_{s,\min}$, the threshold values for TTB (12) and TTS (13) can be determined. In this way, the safety distance is transferred into the time domain and steered by $a_{\mathrm{req},x}$ and $a_{\mathrm{req},y}$. With $a_{\mathrm{req},x}$ and $a_{\mathrm{req},y}$, it is possible to interpret the thresholds and link them to objective or subjective safety and comfort investigations. Now, at a certain $\tau_{b}$, when $d_{0}=d_{b,\min}$, we can claim—if there is sudden maximum braking of the target vehicle—that the ego will need to brake with $a_{\mathrm{req},x}$ to avoid a collision; the same applies for steering.

(12) $$\begin{array}{c} \frac{1}{2}\left(a_{\mathrm{ego},x}-\frac{a_{\mathrm{ego},x}^{2}}{a_{\min,x}}\right)\tau_{b,\mathrm{th}}^{2}+\left(v_{\mathrm{rel}}-\frac{v_{\mathrm{rel}}\;a_{\mathrm{ego},x}}{a_{\min,x}}\right)\tau_{b,\mathrm{th}}-d_{b,\min}-\frac{v_{\mathrm{rel}}^{2}}{2\;a_{\min,x}}=0 \end{array}$$

(13) $$\begin{array}{c} \frac{1}{2}\;a_{\mathrm{ego},x}\;\tau_{s,\mathrm{th}}^{2}+\left(v_{\mathrm{rel}}+a_{\mathrm{ego},x}\;\sqrt{2\;\frac{d_{y}}{a_{\max,y}}}\right)\tau_{s,\mathrm{th}}-d_{s,\min}+v_{\mathrm{rel}}\;\sqrt{2\;\frac{d_{y}}{a_{\max,y}}}=0 \end{array}$$

In the present work, different safety thresholds were defined according to the different criticality values determined by the required accelerations $a_{\mathrm{req},x}$ and $a_{\mathrm{req},y}$, which were obtained from the literature and are provided in Table 2. Driving comfort was taken as the criterion to determine the lowest deceleration threshold level. Several studies, such as [25,26], have correlated the subjective evaluation of driving comfort to objective values, and one of the most significant is acceleration. Taking the relevant literature into account, $-2\;{m/s}^{2}$ was considered as the lowest threshold for longitudinal acceleration. The other extreme is the highest threshold, which corresponds to emergency braking and depends on the current friction coefficient. In this study, we defined the emergency level by −μg. Inbetween these limits and regarding the criticality levels defined below, we defined two intermediate levels based on the partial braking of adaptive cruise control (ACC) [27]. Therefore, the lower intermediate level is $-3\;{m/s}^{2}$ and the higher intermediate level is $-5\;{m/s}^{2}$. Furthermore, the lateral direction ($a_{\mathrm{req},y}$) is determined based on the lane change duration. For a comfortable lane change, we have chosen 6 s, which is an acceptable value based on the driving simulator research in [28]. The intermediate values are an educated guess based on the analysis of the naturalistic driving data derived in [29]. According to [29], the lane change duration varies from 0.7 s to 16.1 s, with a mean of 3.81 s and a standard deviation of 2.03 s. For the first intermediate step, we have chosen 4 s because it fits with the studies conducted in [28,29]. The second intermediate step is 2 s, which is based on the standard deviation presented in [29]. The emergency maneuver is set as previously defined with $a_{\max,y}=\;7$ ${m/s}^{2}$, which also fits the order size of the lower values in [29]. It should be highlighted that the values for lateral acceleration in Table 2 are explicitly derived from subjective studies on lane change and calculated using a simplified equation for the evasion time $t_{\mathrm{ev}}$ (5), where the lateral evasion distance, $d_{y}$, is the lane width 3.5 m. Therefore, different lateral accelerations can be expected if other studies or advanced equations for $t_{\mathrm{ev}}$ are considered.

#### 3.2.2. Criticality Scale

**Figure 4:**
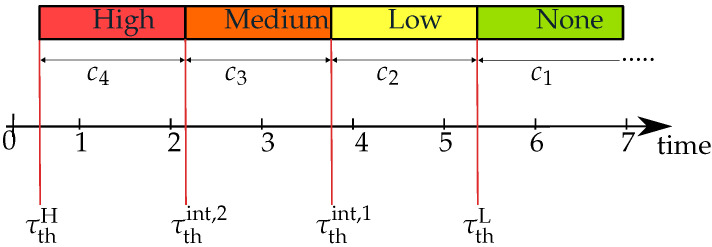
Illustration of the criticality levels.

Based on the VCT, the criticality is quantified with four criticality values, $c_{i}$. This procedure is illustrated in Figure 4, where the index i∈{1,2,3,4} represents the values for a certain criticality level. To calculate those values, the relations from (8), (9), (12) and (13), and the TTRs are needed. Using the longitudinal and lateral acceleration levels from Table 2 as the required deceleration for the safety distance $d_{b,\min}$ and $d_{s,\min}$ in (10) and (11), the distance relations are calculated for the longitudinal and lateral direction in (14).

Longitudinal direction
  $$\begin{array}{cc} d_{b,\min}^{L} & =d_{b,\min}\left(a_{\mathrm{req},x}=-2\;{m/s}^{2}\right)\\ d_{b,\min}^{\mathrm{int},1} & =d_{b,\min}\left(a_{\mathrm{req},x}=-3\;{m/s}^{2}\right)\\ d_{b,\min}^{\mathrm{int},2} & =d_{b,\min}\left(a_{\mathrm{req},x}=-5\;{m/s}^{2}\right)\\ d_{b,\min}^{H} & =d_{b,\min}\left(a_{\mathrm{req},x}=-\mu \;g\right) \end{array}$$
Lateral direction
  (14) $$\begin{array}{cc} d_{s,\min}^{L} & =d_{s,\min}\left(a_{\mathrm{req},y}=0.2\;{m/s}^{2}\right)\\ d_{s,\min}^{\mathrm{int},1} & =d_{s,\min}\left(a_{\mathrm{req},y}=0.5\;{m/s}^{2}\right)\\ d_{s,\min}^{\mathrm{int},2} & =d_{s,\min}\left(a_{\mathrm{req},y}=1.9\;{m/s}^{2}\right)\\ d_{s,\min}^{H} & =d_{s,\min}\left(a_{\mathrm{req},y}=\mu \;g\right) \end{array}$$

The minimum distances $d_{b,\min}^{j}$ and $d_{s,\min}^{j}$ with j∈{L,int,1,int,2,H} from (14) represent the minimum distance between the ego and target vehicle at which evasive maneuver must be performed with corresponding $a_{\mathrm{req},x}$ or $a_{\mathrm{req},y}$ to avoid a collision. Applying these distances in the threshold relation (12) and (13), the VCTs are defined. The VCT levels are calculated according to (15).

TTB ≥ TTS
  $$\begin{array}{cc} \tau_{\mathrm{th}}^{L} & =\tau_{b,\mathrm{th}}\left(d_{b,\min}=d_{b,\min}^{L}\right)\\ \tau_{\mathrm{th}}^{\mathrm{int},1} & =\tau_{b,\mathrm{th}}\left(d_{b,\min}=d_{b,\min}^{\mathrm{int},1}\right)\\ \tau_{\mathrm{th}}^{\mathrm{int},2} & =\tau_{b,\mathrm{th}}\left(d_{b,\min}=d_{b,\min}^{\mathrm{int},2}\right)\\ \tau_{\mathrm{th}}^{H} & =\tau_{b,\mathrm{th}}\left(d_{b,\min}=d_{b,\min}^{H}\right) \end{array}$$
TTB < TTS
  (15) $$\begin{array}{cc} \tau_{\mathrm{th}}^{L} & =\tau_{s,\mathrm{th}}\left(d_{s,\min}=d_{s,\min}^{L}\right)\\ \tau_{\mathrm{th}}^{\mathrm{int},1} & =\tau_{s,\mathrm{th}}\left(d_{s,\min}=d_{s,\min}^{\mathrm{int},1}\right)\\ \tau_{\mathrm{th}}^{\mathrm{int},2} & =\tau_{s,\mathrm{th}}\left(d_{s,\min}=d_{s,\min}^{\mathrm{int},2}\right)\\ \tau_{\mathrm{th}}^{H} & =\tau_{s,\mathrm{th}}\left(d_{s,\min}=d_{s,\min}^{H}\right) \end{array}$$

These threshold values represent four intervals for the TTR. Depending on the range in which the TTR time is, a criticality value according to (16) is determined. The less time available for an evasive maneuver, the more critical the situation is. It is important to notice that threshold intervals are changeable over time and depend on the vehicle states, such as $v_{\mathrm{ego}}$, $a_{\mathrm{ego}}$ and $v_{t}$.

(16) $$c_{i}=\left\{\begin{array}{cc} c_{1}=1,\; & \mathrm{TTR}\geq \tau_{\mathrm{th}}^{L}\\ c_{2}=2,\;\tau_{\mathrm{th}}^{L}> & \mathrm{TTR}\geq \tau_{\mathrm{th}}^{\mathrm{int},1}\\ c_{3}=3,\;\tau_{\mathrm{th}}^{\mathrm{int},1}> & \mathrm{TTR}\geq \tau_{\mathrm{th}}^{\mathrm{int},2}\\ c_{4}=4,\;\tau_{\mathrm{th}}^{\mathrm{int},2}> & \mathrm{TTR}\geq \tau_{\mathrm{th}}^{H} \end{array}\right.$$

### 3.3. Criticality Assessment Method

To assess the overall criticality of the scenario we are combining the criticality definition from (16) with the concept of the fictive vehicles depicted in Figure 3. Considering the three-lane configuration from Figure 3, it is possible to place two fictive vehicles as the condition of unoccupied adjacent lanes is satisfied. For each of those fictive vehicles, as well as for ego, the criticality values according to (16) are determined. However, for the fictive vehicle, only braking as an evasive maneuver is considered which leads to TTR=TTB, while for the ego, TTR = max{TTB, TTS}. Now, the overall criticality $c_{s}$ is determined by (17). This equation takes mean criticality values calculated between ego ($c_{\mathrm{ego}}$) and each fictive vehicle ($c_{\mathrm{ego},f}^{r}$ and $c_{\mathrm{ego},f}^{l}$). The mean values are rounded to the next higher value, and the lower is chosen as the overall criticality. In the case of equal mean values for the left and right lanes, the one with a higher TTR is taken.

(17) $$\begin{array}{c} c_{s}=\min\left\{\left\lceil \frac{c_{\mathrm{ego}}+c_{\mathrm{ego},f}^{r}}{2}\right\rceil ,\;\;\left\lceil \frac{c_{\mathrm{ego}}+c_{\mathrm{ego},f}^{l}}{2}\right\rceil \right\} \end{array}$$

Additionally, if a trailing vehicle exists, the time gaps $\tau_{\mathrm{gap},l}$ and/or $\tau_{\mathrm{gap},r}$ are calculated and considered for the criticality assessment. The time gap $\tau_{\mathrm{gap},l}$ represents a time gap between the trailing vehicle at the left side and $\tau_{\mathrm{gap},r}$ at the right side. The time gap itself represents the time interval between the fictive ego vehicles ${\mathrm{EGO}}_{f}^{l}$ and ${\mathrm{EGO}}_{f}^{r}$ and the trailing vehicles $T_{l}$ and $T_{r}$. The calculation of the time gaps for the left and right sight are presented in

(18) $$\tau_{\mathrm{gap},l}=\frac{\left(s_{T_{l}}-s_{{\mathrm{ego}}_{f}^{l}}\right)}{v_{T_{l}}}\;,$$

(19) $$\tau_{\mathrm{gap},r}=\frac{\left(s_{T_{r}}-s_{{\mathrm{ego}}_{f}^{r}}\right)}{v_{T_{r}}}$$

with $s_{T_{l}}$ and $s_{T_{r}}$ being the trailing vehicle positions; $v_{T_{l}}$ and $v_{T_{r}}$ being the trailing vehicle velocities; $s_{{\mathrm{ego}}_{f}^{l}}$ and $s_{{\mathrm{ego}}_{f}^{r}}$ being the fictive ego vehicle positions. Here, $\tau_{\mathrm{gap},r}^{\mathrm{th}}$ and $\tau_{\mathrm{gap},l}^{\mathrm{th}}$ represent the threshold values for the time gap. If the time gape values are lower than these thresholds, the possibility of steering will not be considered for the overall assessment. In this case, TTR is set to be TTB.

## 4. Simulation Results

**Figure 5:**
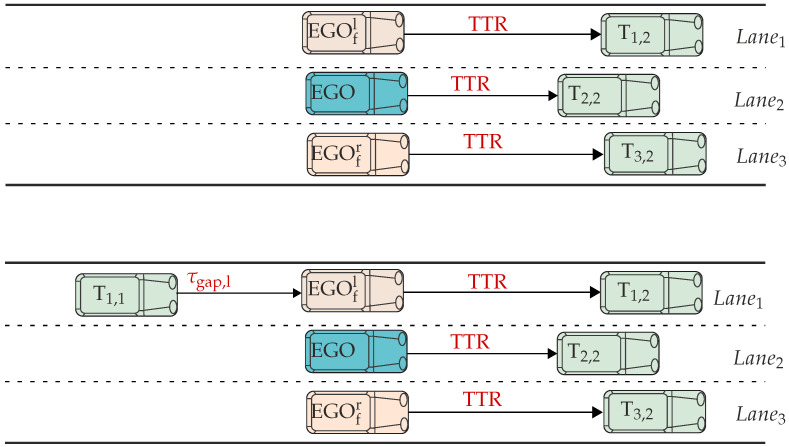
Graphical representation of test scenarios.

For demonstrating the introduced assessment method, two exemplary test scenarios shown in Figure 5 are taken into account. In the first scenario, the ego vehicle is approaching the target vehicle $T_{2,2}$ in lane 2, while vehicles $T_{1,2}$ and $T_{3,2}$ are placed adjacent to $T_{2,2}$. In the second scenario, the trailing $T_{1,1}$ is added. Threshold for the time gap ($\tau_{\mathrm{gap},l}^{\mathrm{th}}$) between $T_{1,1}$ and ${\mathrm{EGO}}_{f}^{l}$ is set to 3 s. This means that, if $\tau_{\mathrm{gap},l}^{\mathrm{th}}\leq 3\;$, then the ego cannot consider steering in lane 1. Scenarios are created and simulated within the simulation software CarMaker from IPG Automotive, and the initial positions and velocities of vehicles are given in Table 3.

**Figure 6:**
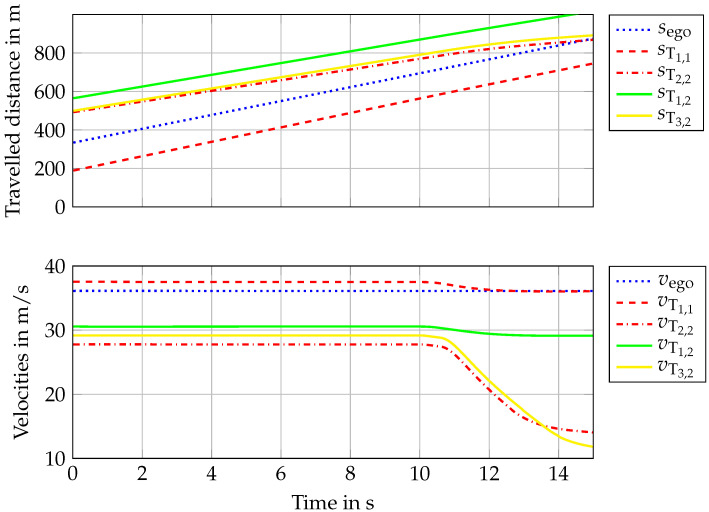
Distances traveled and velocities in the tested two scenarios.

Figure 6 shows the traveled distance and velocities of vehicles; while the ego vehicle keeps its speed constant, the target vehicles reduce their speed after 10 s to the defined velocities. Finally, scenarios will lead to the collision of the ego with $T_{2,2}$, as the ego vehicle does not brake and has no assistance functions. Collision is not mitigated on purpose, in order to demonstrate how the criticality of the scenario changes as the ego approaches $T_{2,2}$.

**Figure 7:**
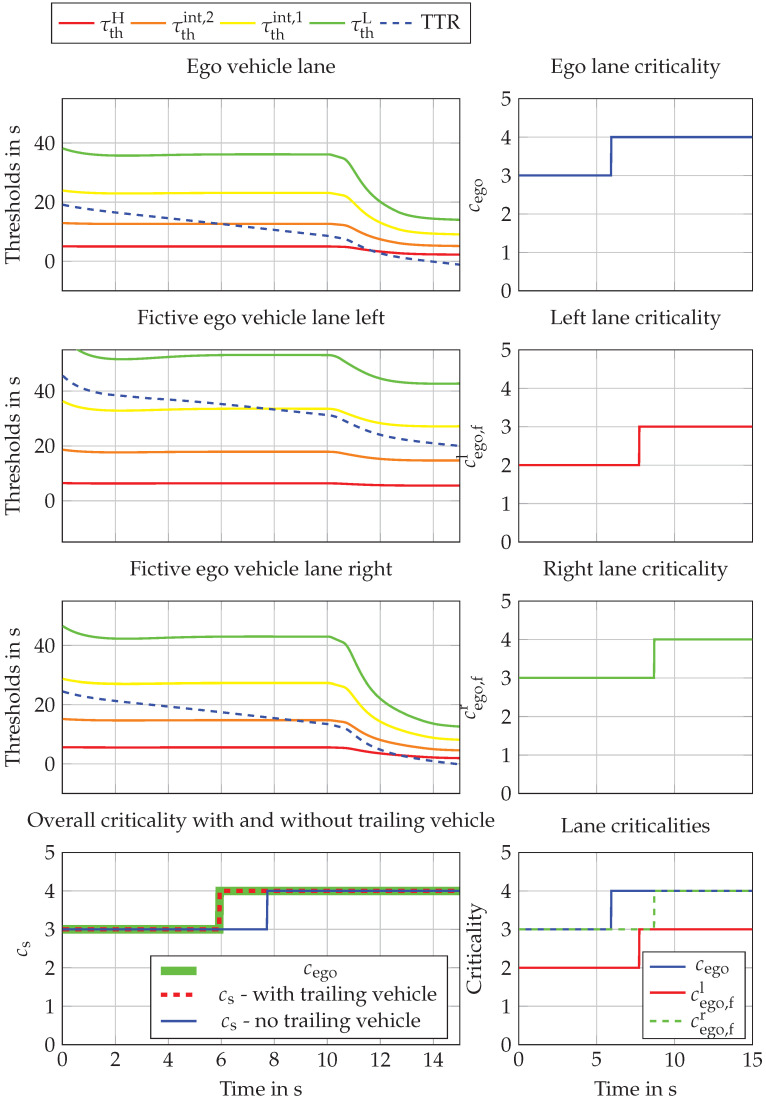
Criticality assessment results for the two introduced scenarios.

Velocity reductions are different for each lane which cause different criticality values, and this behavior is shown in Figure 7. It can be seen that the criticality of the ego lane has the longest criticality phase with a criticality value of 4 (high criticality). This is because of the aggressive speed-reduction maneuver of the front vehicle, $T_{2,2}$. The right lane has a slightly lower criticality, and the left one has the lowest criticality over time. Because the left lane has a criticality of 2 (low criticality) until 7.72 s, it represents a potentially avoidable situation for the ego vehicle in this time interval. Therefore, the overall criticality $c_{s}$ for the ego vehicle is one level lower between 5.92 s and 7.62 s, as shown in Figure 7. This is only valid up to 7.62 s, where the ego vehicle comes closer to the vehicle $T_{2,2}$ and the potential evasive maneuver space becomes smaller. The last point where the ego vehicle could make evasive maneuver is at the point where the TTR time intersects the $\tau_{\mathrm{th}}^{H}$ at 11.6 s. After that, a collision cannot be avoided anymore.

**Figure 8:**
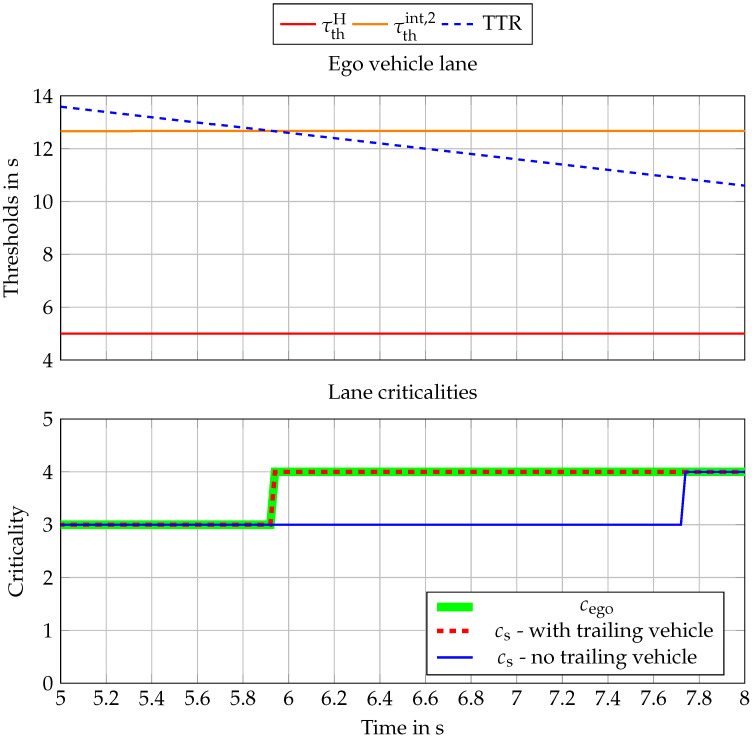
Criticality assessment results for the time frame between 5 s and 8 s.

The whole scenario behaves slightly differently when a trailing vehicle is considered. The small time gap between the $T_{1,1}$ and ${\mathrm{EGO}}_{f}^{l}$ eliminates the possibility of ego to steer in the left lane so the total criticality rises to the maximum criticality for the time interval 5–8 s, see Figure 8.

In summary, these two examples demonstrate the functionality of the criticality assessment method for the assumptions made to calculate the TTR and the chosen VCT. In addition, these particular examples were chosen to show the potential of the method by considering and analyzing the influences of the ego vehicles surrounding for the criticality calculation.

## 5. Conclusions

In the current paper, we introduced a criticality assessment method for ADS. The novelty of the method lies in the introduction of fictive ego vehicles from the surrounding of the ego. In order to determine the overall criticality, the collision time reserve as TTR metric was introduced and evaluated using four criticality levels. The criticality levels are determined using variable threshold values, which are converted into the time domain from minimal safety distance metrics and acceleration values. Considered acceleration values present a linkage between objective investigation and threshold values, while the derived VCT fills the research gap by taking into account the kinematic relations between vehicles, and thus variable nature of the criticality. To derive feasible and intelligible method, certain assumption has been made. The method is derived for straight highway roads, while formulation assumes constant velocity of a target vehicle.

Finally, the method is demonstrated on two highway scenarios that defer in the presence of a trailing vehicle. The results show higher criticality of scenarios in which trailing vehicles obstruct the adjacent lane of the ego. This narrowed down the alternative options for the ego and increased the overall criticality. The potential and advantage of the proposed criticality assessment method reside in simplified consideration of the surrounding ego vehicles and the evaluation of possible evasive actions; in addition to the criticality assessment, it could be used for decision-making applications. For future works, the proposed criticality metric and threshold calculation will be applied in a wide range of scenarios, while the mathematical formulation will be extend to cover considered assumptions and to make realistic lane change models for steering maneuvers.

## Figures and Tables

**Table 2:** Acceleration levels for lateral and longitudinal movement.

Acceleration Levels	$a_{\mathrm{req},x}$	$a_{\mathrm{req},y}$
Comfort	$-2\;{m/s}^{2}$	$0.2\;{m/s}^{2}$
Intermediate 1	$-3\;{m/s}^{2}$	$0.5\;{m/s}^{2}$
Intermediate 2	$-5\;{m/s}^{2}$	$1.9\;{m/s}^{2}$
Emergency	−μg	$7\;{m/s}^{2}$

**Table 3:** Initial states and parameters for the test scenarios.

Scenario Description				
	Start position on the lane in m	Start velocity in km/h	Speed reduction time in s	Desired speed reduction in km/h
**Scenario 1**	Trailing vehicle is not considered			
EGO	50	130	None	None
$T_{1,2}$	250	105	15	90
$T_{2,2}$	250	100	15	50
$T_{2,3}$	250	105	15	70
**Scenario 2**	Trailing vehicle is considered.			
Trailing vehicle	0	130	15	100

## Data Availability

Not applicable.

## Abbreviations

ADS	automated driving systems
SCS	safety-critical scenario
TTC	time to collision
TTR	time to react
TTB	time to brake
TTS	time to steer
TTK	time to kickdown
TTx	time to x
LPTB	last point to brake
LPTS	last point to steer
VCT	variable criticality threshold
